# Supplementary material for: Molecular engineering of a cryptic epitope in Spike RBD improves manufacturability and neutralizing breadth against SARS-CoV-2 variants
Source: Vaccine. 2023 Jan 27;41(5):1108–18. doi: 10.1016/j.vaccine.2022.12.062 (PMC9797419; doi:10.1016/j.vaccine.2022.12.062)

**
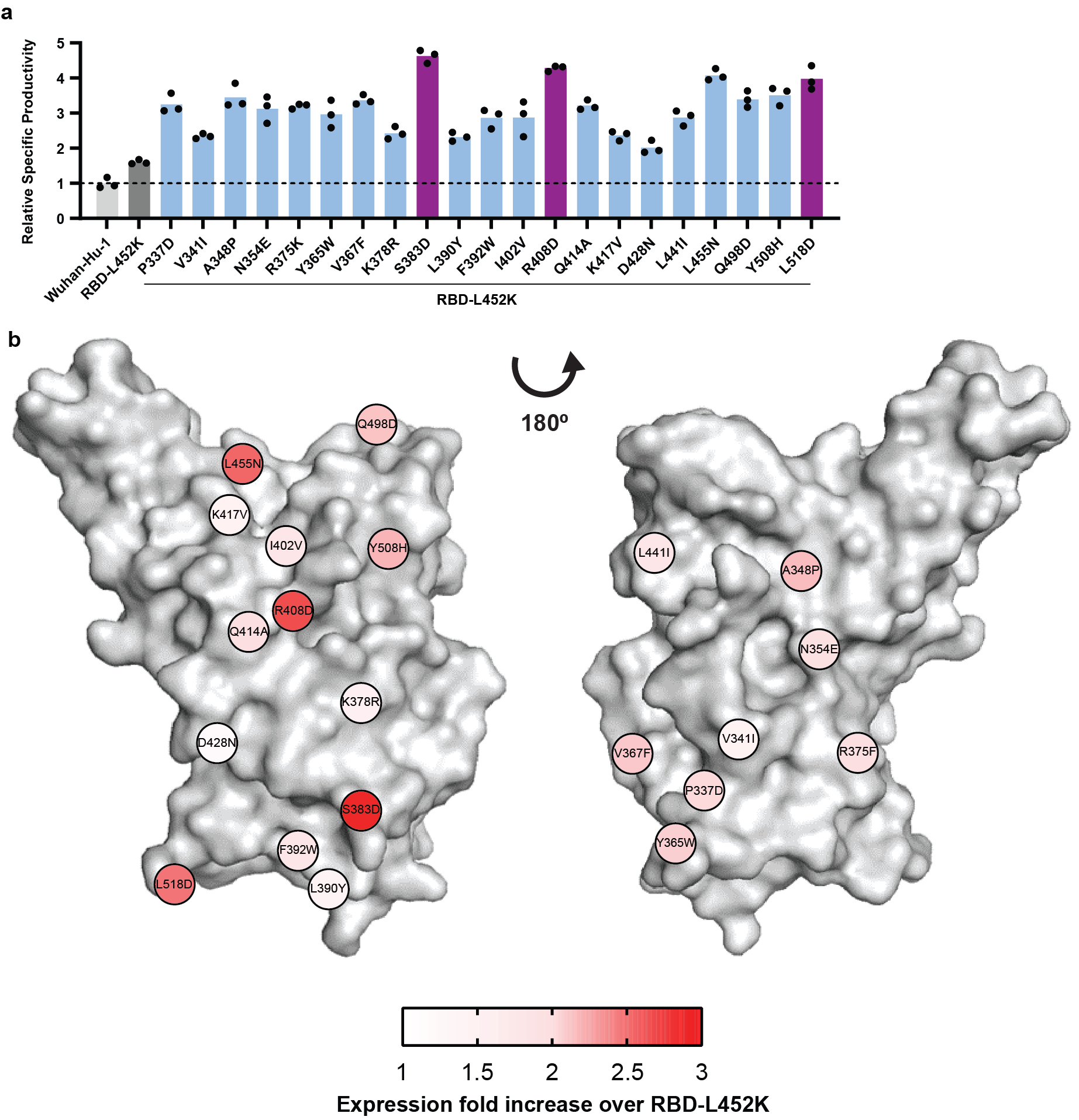
**

**Supplementary Fig. S1 Expression titer of 21 mutations coupled with RBD-L452K**

(**a**) Titer of mutated RBD secretion in 3mL plate cultures, measured by reverse-phase liquid chromatography. Bars represent mean values. (**b**) Localization of mutations coupled with RBD-L452K on RBD surface. Mutations are color-coded according to their expression fold increase over RBD-L452K.

**
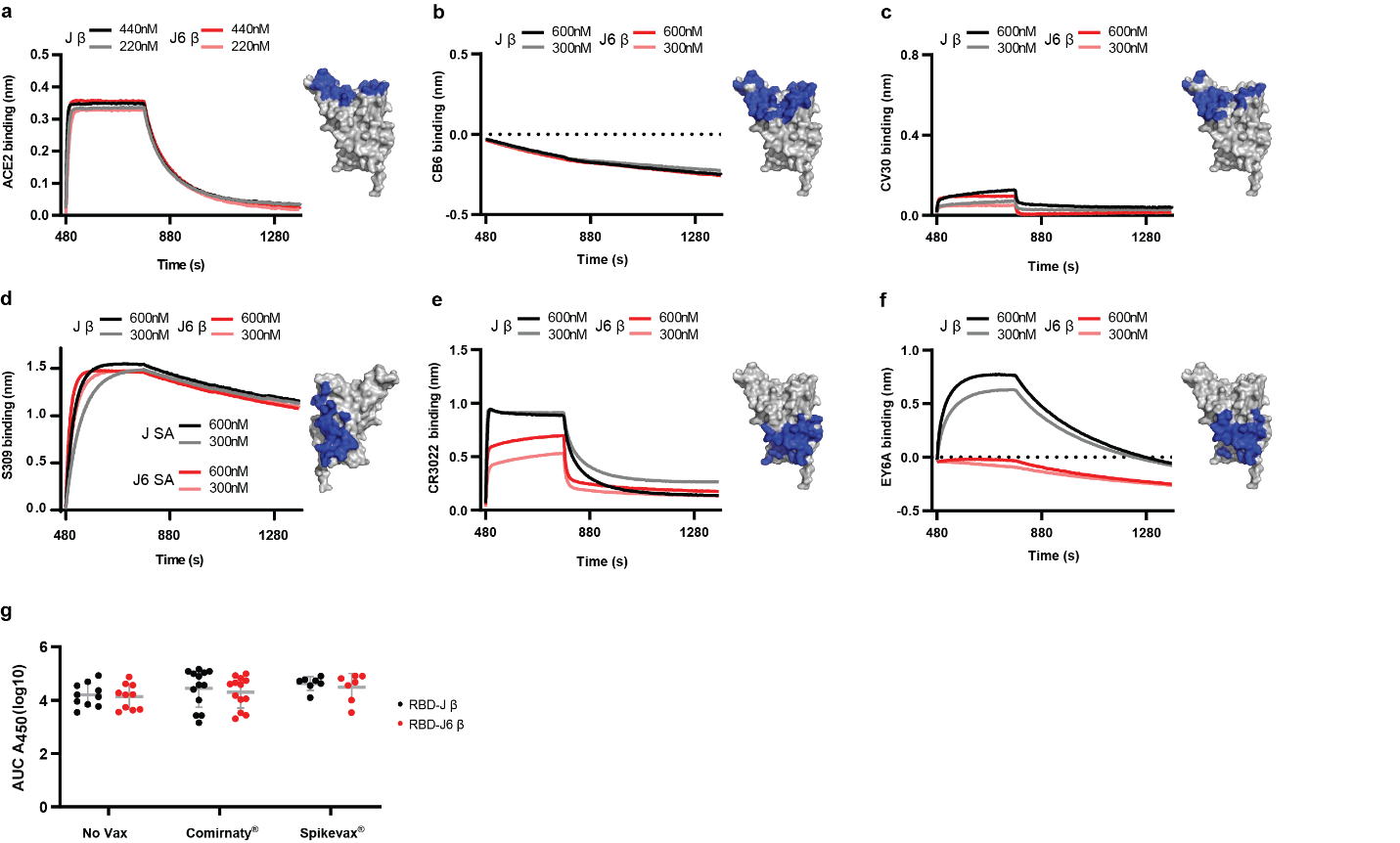
**

**Supplementary Fig. S2 Antigenic characterization of RBD-J β and RBD-J6 β**

Binding of purified RBD to (**a**) human ACE2-Fc fusion protein, (**b**) CB6, class I neutralizing antibody, (**c**) CV30, class I neutralizing antibody, (**d**) S309, class III neutralizing antibody, (**e**) CR3022, class IV neutralizing antibody, and (**f**) EY6A, class IV neutralizing antibody by biolayer interferometry. Blue regions on the RBD structure indicate target binding epitopes. (**g**) Area under the curve for antibody binding titers of Delta variant breakthrough cases’ convalescent sera from unvaccinated, Comirnaty^®^ (Pfizer-BioNTech) vaccinated, and Spikevax^®^ (Moderna) vaccinated cohorts. Gray bars represent mean and standard deviation of sample set.

**
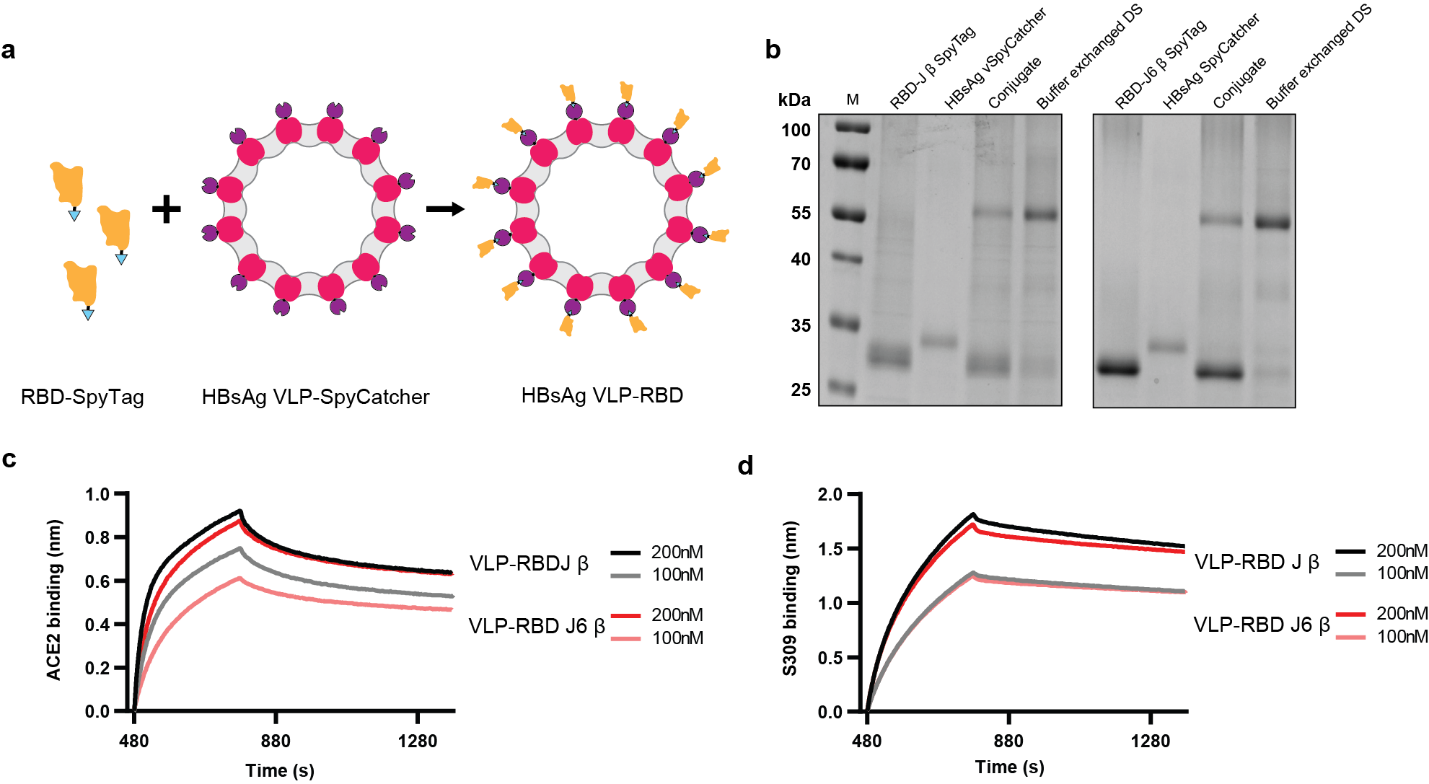
**

**Supplementary Fig. S3 Design and characterization of VLP-RBD drug product**

(**a**) Schematic of RBD-SpyTag conjugation onto HBsAg-SpyCatcher VLP. (b) Reduced SDS-PAGE analysis of conjugated RBD-J β VLP (left) and RBD-J6 β VLP (right). (**c-d**) Binding of VLP-RBD to (**c**) human ACE2-Fc fusion protein and (**d**) S309, class III neutralizing antibody, measured by biolayer interferometry.

**
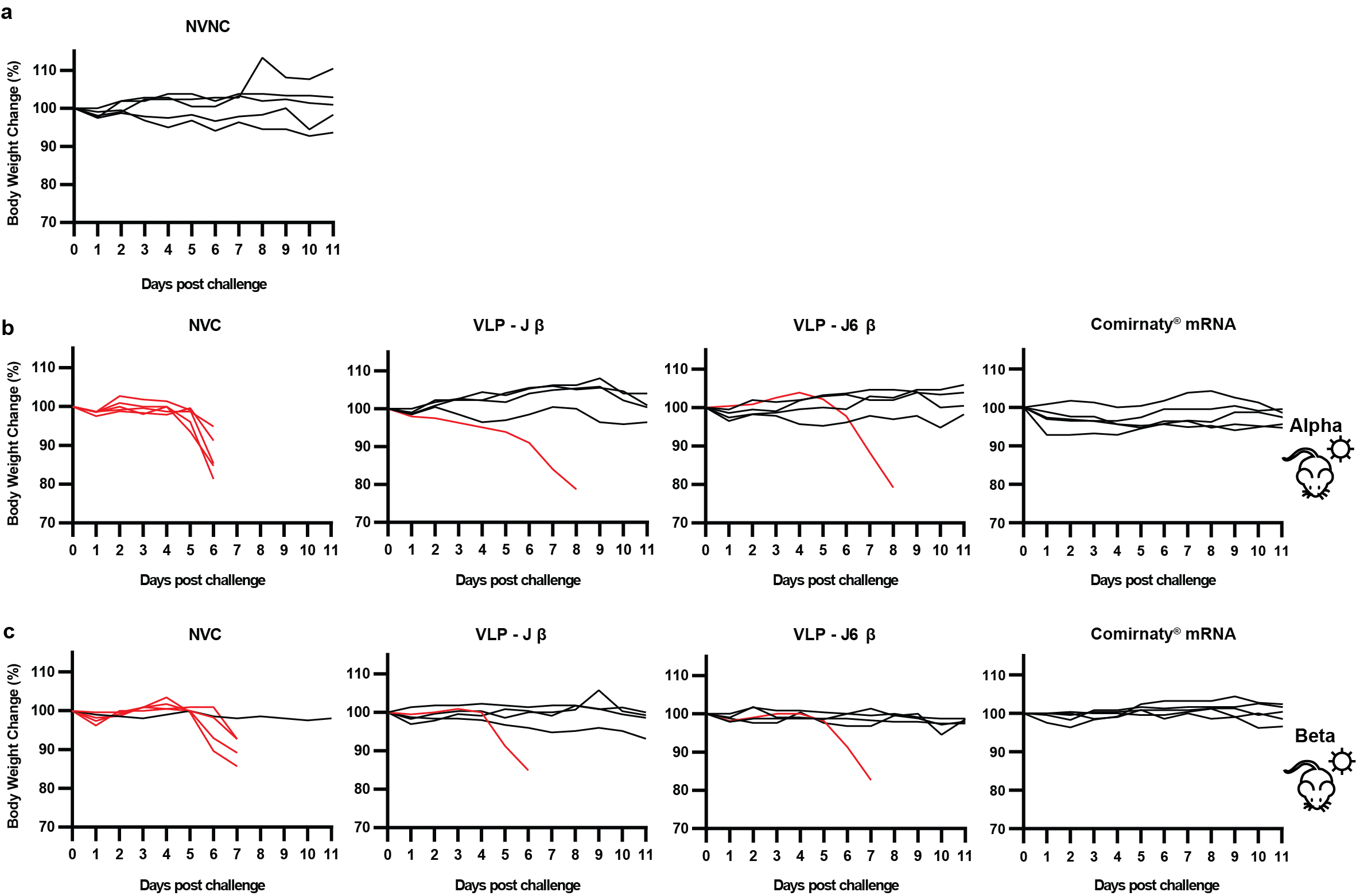
**

**Supplementary Fig. S4 Body weight change during SARS-CoV-2 challenge**

Body weight tracking of (**a**) non-vaccinated, non-challenged (NVNC) mice, (**b**) mice challenged with SARS-CoV-2 Alpha variant, and (**c**) mice challenged with SARS-CoV-2 Beta variant. Body weight of euthanized mice are labeled in red. NVC – non-vaccinated, challenged mice.

**
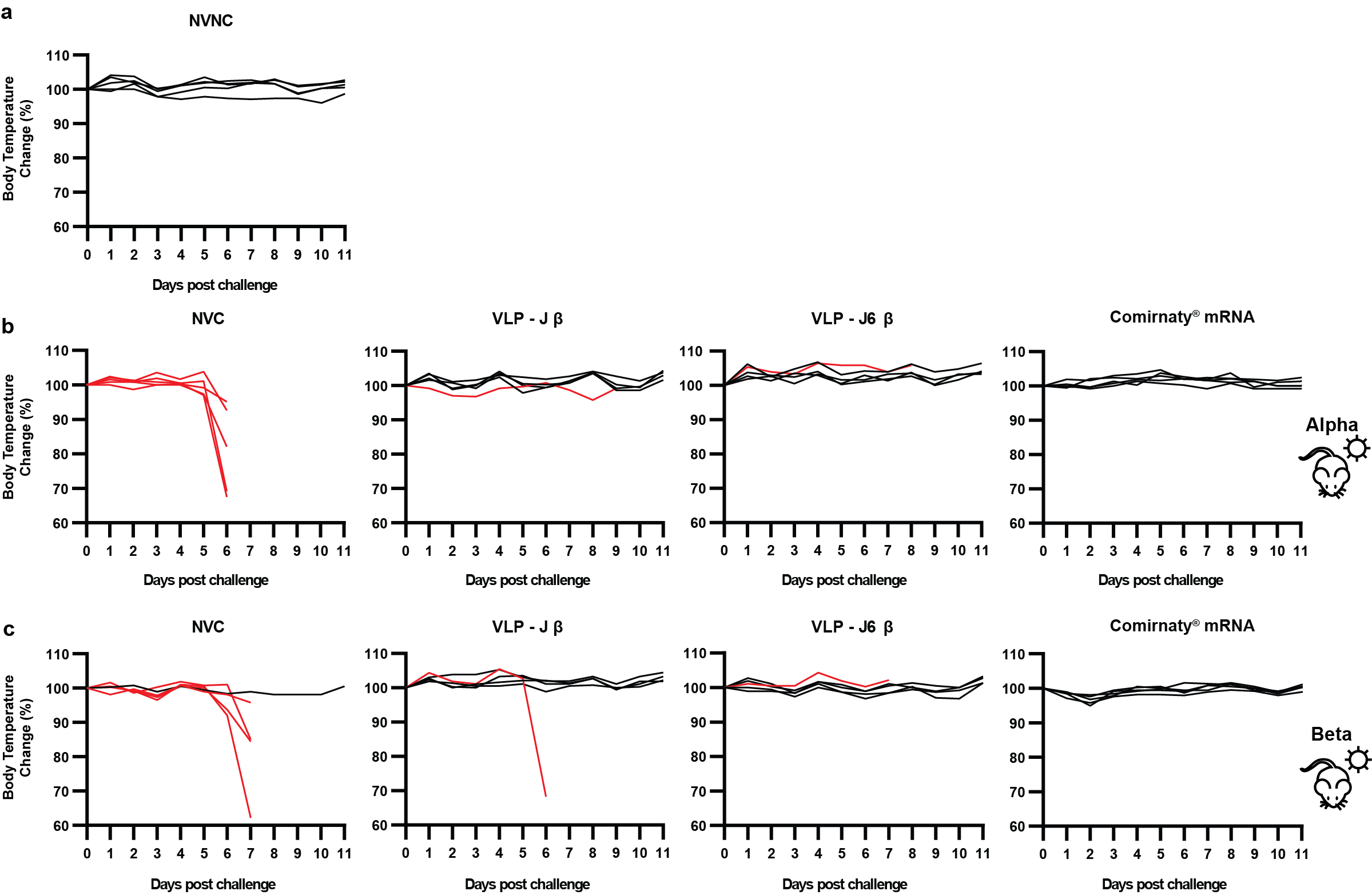
**

**Supplementary Fig. S5 Body temperature change during SARS-CoV-2 challenge**

Body temperature tracking of (**a**) non-vaccinated, non-challenged (NVNC) mice, (**b**) mice challenged with SARS-CoV-2 Alpha variant, and (**c**) mice challenged with SARS-CoV-2 Beta variant. Body temperature of euthanized mice are labeled in red. NVC – non-vaccinated, challenged mice.


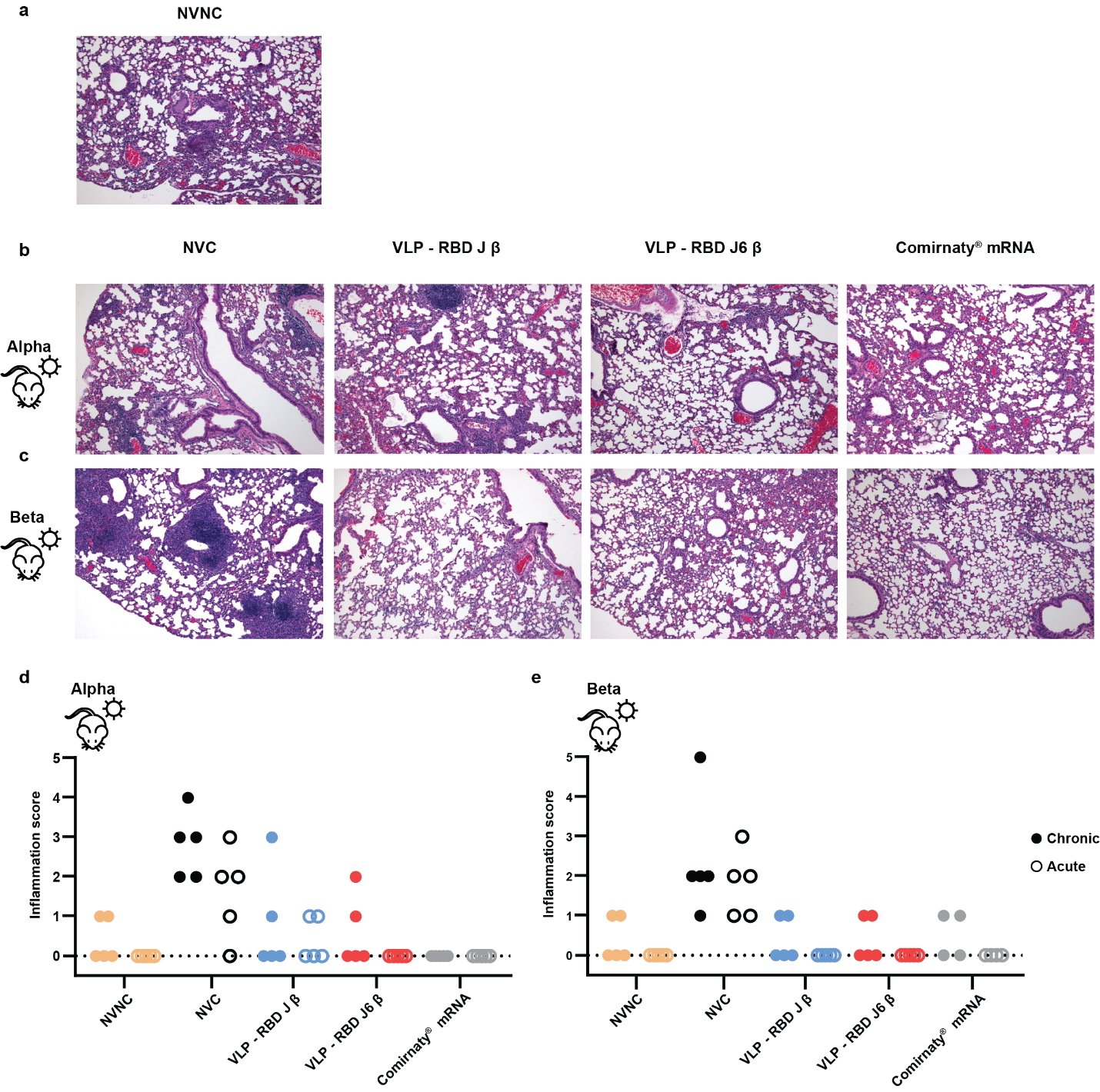


**Supplementary Fig. S6 Histopathological analysis of lung tissue from SARS-CoV-2 challenged mice**

Hematoxylin and eosin staining of lung tissue from non-vaccinated, non-challenged (NVNC) (**a**), SARS-CoV-2 Alpha (**b**) and Beta (**c**) challenged mice. Images presented at a 100x magnification. Chronic and acute inflammation scores of lungs from NVNC mice and SARS-CoV-2 Alpha (**d**) and Beta (**e**) variant challenged mice.


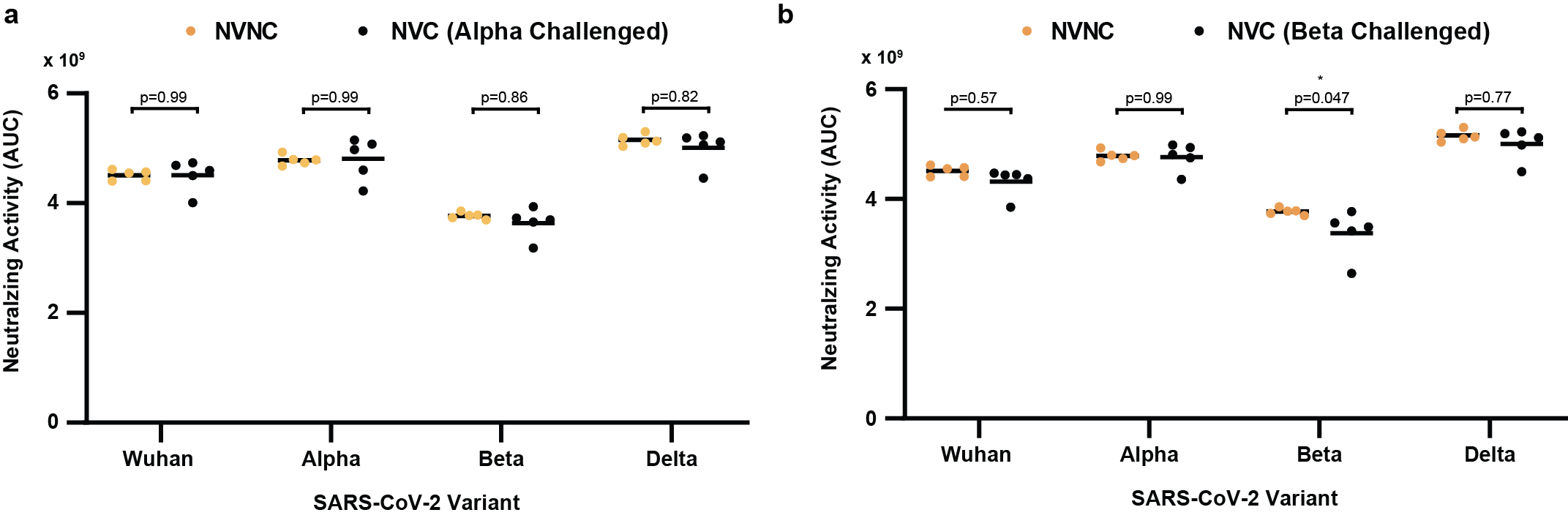


**Supplementary Fig. S7** **Comparison of neutralizing activity of sera from non-vaccinated, no challenge (NVNC) and non-vaccinated, challenged (NVC) mice.**

MSD ACE2 neutralizing activity of VOC RBDs of post Alpha (**a**) and Beta (**b**) challenge sera against ancestral SARS-CoV-2 and variants. Points represent area under the curve of a serum dilution curve. Lower AUC indicates higher serum neutralizing activity. Black bars represent mean values. Statistical significance was determined by ordinary two-way ANOVA, using Sidak’s multiple comparison test (**p*<0.05).

**Supplementary Table 1. Statistical analysis of variant specific IgG titers after dose #1 (week 2).** Significance was determined using Two-way Ordinary ANOVA using Tukey’s multiple comparison method (**p*<0.05, ***p*<0.01, ****p*<0.001, *****p*<0.001).
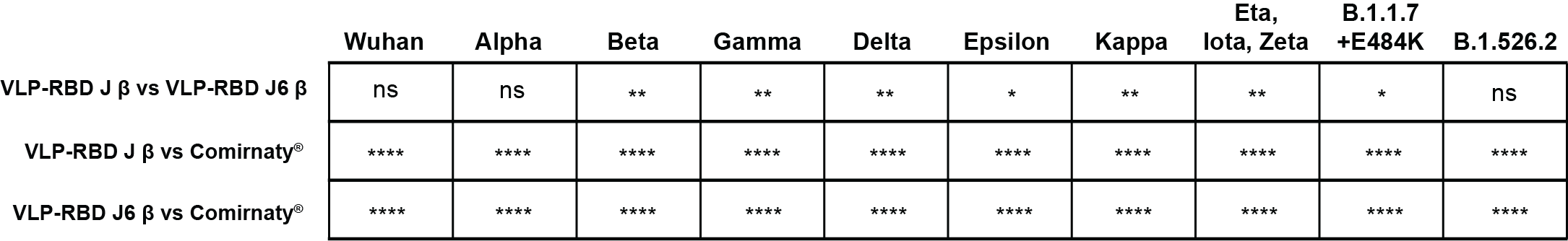


**Supplementary Table 2. Statistical analysis of variant specific IgG titers after dose #3 (week 7).** Significance was determined using Two-way Ordinary ANOVA using Tukey’s multiple comparison method (**p*<0.05, ***p*<0.01, ****p*<0.001, *****p*<0.001).


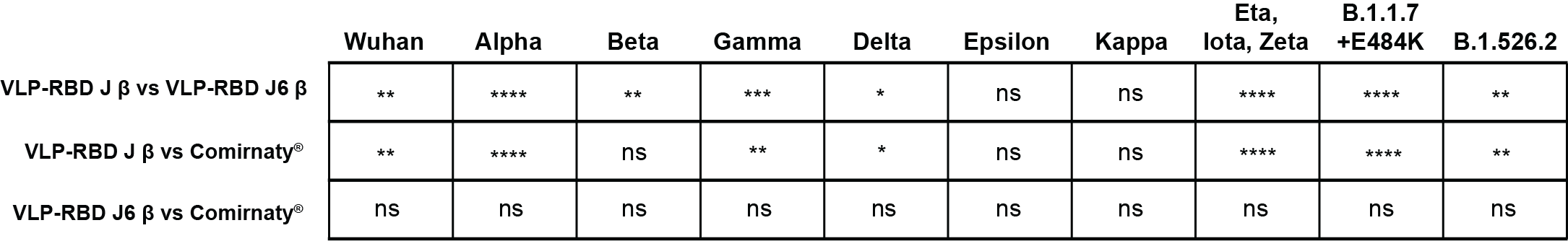

Supplement: Supplementary data 1 [file mmc1.docx]
